# Sex‐opposed inflammatory effects of 27‐hydroxycholesterol are mediated via differences in estrogen signaling

**DOI:** 10.1002/path.5477

**Published:** 2020-07-07

**Authors:** Tom Houben, Albert V Bitorina, Yvonne Oligschlaeger, Mike LJ Jeurissen, Sander Rensen, S Eleonore Köhler, Marit Westerterp, Dieter Lütjohann, Jan Theys, Andrea Romano, Jogchum Plat, Ronit Shiri‐Sverdlov

**Affiliations:** ^1^ Department of Molecular Genetics, School of Nutrition & Translational Research Maastricht (NUTRIM) Maastricht University Maastricht The Netherlands; ^2^ Department of Surgery, School of Nutrition & Translational Research Maastricht (NUTRIM) Maastricht University Maastricht The Netherlands; ^3^ Department of Anatomy & Embryology, School of Nutrition & Translational Research Maastricht (NUTRIM) Maastricht University Maastricht The Netherlands; ^4^ Department of Pediatrics, Section Molecular Genetics University of Groningen, University Medical Center Groningen Groningen The Netherlands; ^5^ Institute of Clinical Chemistry and Clinical Pharmacology University of Bonn Bonn Germany; ^6^ Department of Precision Medicine, GROW School for Oncology and Developmental Biology Maastricht University Maastricht The Netherlands; ^7^ Department of Obstetrics & Gynaecology, School for Oncology and Developmental Biology Maastricht University Maastricht The Netherlands; ^8^ Department of Nutrition and Movement Sciences, School of Nutrition & Translational Research Maastricht (NUTRIM) Maastricht University Maastricht The Netherlands

**Keywords:** 27‐hydroxycholesterol, inflammation, sex differences, estrogen

## Abstract

Despite the increased awareness of differences in the inflammatory response between men and women, only limited research has focused on the biological factors underlying these sex differences. The cholesterol derivative 27‐hydroxycholesterol (27HC) has been shown to have opposite inflammatory effects in independent experiments using mouse models of atherosclerosis and non‐alcoholic steatohepatitis (NASH), pathologies characterized by cholesterol‐induced inflammation. As the sex of mice in these *in vivo* models differed, we hypothesized that 27HC exerts opposite inflammatory effects in males compared to females. To explore whether the sex‐opposed inflammatory effects of 27HC translated to humans, plasma 27HC levels were measured and correlated with hepatic inflammatory parameters in obese individuals. To investigate whether 27HC exerts sex‐opposed effects on inflammation, we injected 27HC into female and male Niemann–Pick disease type C1 mice (*Npc1* ^*nih*^) that were used as an extreme model of cholesterol‐induced inflammation. Finally, the involvement of estrogen signaling in this mechanism was studied in bone marrow‐derived macrophages (BMDMs) that were treated with 27HC and 17**β**‐estradiol (E2). Plasma 27HC levels showed opposite correlations with hepatic inflammatory indicators between female and male obese individuals. Likewise, hepatic 27HC levels oppositely correlated between female and male *Npc1* ^*nih*^ mice. Twenty‐seven hydroxycholesterol injections reduced hepatic inflammation in female *Npc1* ^*nih*^ mice in contrast to male *Npc1* ^*nih*^ mice, which showed increased hepatic inflammation after 27HC injections. Furthermore, 27HC administration also oppositely affected inflammation in female and male BMDMs cultured in E2‐enriched medium. Remarkably, female BMDMs showed higher ERα expression compared to male BMDMs. Our findings identify that the sex‐opposed inflammatory effects of 27HC are E2‐dependent and are potentially related to differences in ER**α** expression between females and males. Hence, the individual’s sex needs to be taken into account when 27HC is employed as a therapeutic tool as well as in macrophage estrogen research in general. © 2020 The Authors. *The Journal of Pathology* published by John Wiley & Sons Ltd on behalf of Pathological Society of Great Britain and Ireland.

## Introduction

An increasing amount of evidence has pointed towards sex differences influencing physiological processes, including inflammatory responses [1, 2, 3, 4, 5]. In spite of these physiological differences between men and women, only limited research has focused on the biological factors underlying these differences. As gender‐based prevention and therapy, as opposed to the ‘one‐size‐fits all’ approach, is expected to benefit both male and female patients [5], it is essential to improve our understanding on these underlying biological factors involved with sex differences.

Metabolically‐induced inflammatory responses (referred to as metabolic inflammation or metaflammation) such as cholesterol‐induced activation of macrophages [6, 7] have been generally accepted as a cause for the chronic low‐grade inflammatory state observed in obese patients [8]. Metaflammation is also responsible for the steep increase in the amount of obese individuals suffering from hepatic inflammation [9], identifying the liver as one of the most sensitive organs for cholesterol‐ and lipid‐induced metaflammation [10]. Relevantly, cholesterol‐derived lipids known as oxysterols have been linked to obesity‐associated inflammatory conditions [11, 12]. The most abundant oxysterol in the human body is 27‐hydroxycholesterol (27HC) [13], which is generated via enzymatic oxidation of cholesterol by sterol 27‐hydroxylase (CYP27A1) [14]. Due to its cholesterol‐expelling effects [15], 27HC has been investigated as a therapeutic agent in atherosclerosis and non‐alcoholic steatohepatitis (NASH), pathologies characterized by cholesterol‐induced macrophage activation leading to inflammation [7, 16, 17, 18, 19]. Whereas hematopoietic overexpression of *Cyp27a1* [20] and subcutaneous administration of 27HC in female hyperlipidemic mice [21] resulted in reduced hepatic inflammation, subcutaneous injections of 27HC in a comparable male hyperlipidemic mouse model paradoxically elevated atherosclerosis via increasing pro‐inflammatory processes [22]. These results suggest that sex differences may underlie the opposite effects of 27HC on cholesterol‐induced inflammation.

Based on these findings, we hypothesized that under cholesterol‐induced inflammatory conditions, 27HC exerts sex‐opposed inflammatory effects. Firstly, we found in a cohort of obese individuals that higher plasma 27HC levels in females corresponded with lower hepatic inflammatory indicators, while the opposite was observed in males. Similar sex‐opposed inflammatory effects of 27HC were observed in Niemann–Pick nih mice (*Npc1* ^*nih*^), here used as an extreme model mimicking cholesterol‐induced inflammation [23]. Finally, *in vitro* experiments with murine bone marrow‐derived macrophages (BMDMs) provided evidence that the sex‐opposed inflammatory effect of 27HC is E2‐dependent and is potentially related to differences in ERα expression between females and males. Altogether, these data demonstrate for the first time the sex‐opposed effects of 27HC on inflammation. Hence, the individual’s sex needs to be taken into account when 27HC is employed as a therapeutic tool.

## Materials and methods

### Human subjects

The study was carried out in accordance with the approved guidelines by the Medical Ethical Committees of both the Maastricht University Medical Centre and the Atrium Medical Centre Parkstad and conducted in accordance with the revised version of the Declaration of Helsinki (October 2008, Seoul) [24]. Written informed consent was obtained from all the subjects.

The Maastricht cohort included obese adult individuals (34; 21 females and 13 males) undergoing bariatric surgery at the Maastricht University Medical Center or at the Atrium Medical Center Parkstad, Heerlen, The Netherlands. Population characteristics are represented in supplementary material, Table S1A. NASH was determined by evaluation of liver biopsies based on Brunt’s criteria by a trained pathologist. Exclusion criteria, the procedure of obtaining liver biopsies, and blood sampling of this cohort have been previously described [25, 26]. Determination of plasma 27HC levels was performed as described previously [21].

### Mice, diet, and treatment

Experiments were performed according to the Dutch regulation and approved by the Committee for Animal Welfare of Maastricht University. *Npc1* ^*nih*^ mice were housed under standard conditions and were given free access to food and water. *Npc1* ^*nih*^ mice (a kind gift from Professor Dr Lieberman from University of Michigan Medical School) were derived from heterozygous founders (C57BL/6‐*Npc1* ^*nih*^). In the first *in vivo* experiment, 7‐week‐old female (*n* = 4) and male (*n* = 4) *Npc1* ^*nih*^ mice were given a normal chow diet and sacrificed. In the second *in vivo* experiment, 2‐week‐old female (*n* = 5 per group) and male (*n* = 5 per group) *Npc1* ^*nih*^ mice were injected daily with 27‐hydroxycholesterol (27HC; 40 mg/kg) or vehicle (2‐hydroxypropyl‐β‐cyclodextrin 18%) for 21 weeks and given a normal chow diet. Tissue specimens were isolated and snap‐frozen in liquid nitrogen and stored at −80 °C or fixed in 4% formaldehyde/PBS. The collection of blood and hepatic and bile oxysterols was carried out as described previously [19, 27, 28]. Product information is provided in supplementary material, Table S1.

### RNA isolation/cDNA synthesis/RT‐qPCR

Total RNA was isolated from mouse liver tissues or cultured bone marrow‐derived macrophages (BMDMs). For liver tissues, homogenization was achieved by adding 1.0 ml of glass beads and 1.0 ml of Tri Reagent (Sigma Aldrich, St Louis, MO, USA) to the frozen liver tissues in a closed tube for 30 s at 4800 rpm. For BMDMs, 350 μl of Tri Reagent was added to the well, after which BMDMs were transferred to a tube. After addition of chloroform (200 μl for liver tissues and 100 μl for BMDMs) and centrifugation, the aqueous phase was transferred to a fresh tube. Isopropanol (0.5 ml) was added and following another centrifugation step, RNA was pelleted and then washed by adding 1.0 ml of 70% ethanol. After a further centrifugation step, the supernatant was removed and the pellet was dissolved in an appropriate volume of DEPC sterile H_2_O. All materials used were RNase‐free and samples were placed on ice during the procedures. Afterwards, the concentration and quality of RNA were determined using a NanoDrop ND‐1000 spectrophotometer.

Total RNA (500 ng for liver and 300 ng for BMDMs) was reverse‐transcribed into complementary DNA (cDNA) using an iScript cDNA synthesis kit according to the manufacturer’s instructions. Changes in the expression of the indicated genes were determined by qPCR using 10 ng of cDNA on Bio‐Rad MyIQ with IQ5 v2.1 software (ABI 7900; Applied Biosystems, Foster City, CA, USA) using IQ SensiMix SYBR master mix with fluorescein (Bioline, London, UK). *Cyclophilin A* (*PpiA*), *ribosomal protein S12* (*Rps12*), and *Actb* were used as reference genes to standardize for the amount of cDNA. By using the default settings in primer Express version 2.0 (Applied Biosystems), primer sets for the selected genes were developed and are available in supplementary material, Table S2. RT‐qPCR data were analyzed according to the relative standard curve method.

### Immunohistochemistry

Frozen liver sections (7 μm) were fixed in acetone and subsequently endogenous peroxidase was blocked by incubation with a 1:400 dilution of 30% H_2_O_2_ for 5 min. Prior to incubation with the first antibody, slides were incubated with 4% new‐born calf serum (Bodinco, Alkmaar, The Netherlands)/1× PBS + 1:5 Avidin D Block solution (Vector Laboratories, Burlingame, CA, USA). Primary antibodies were used to detect infiltrated macrophages and neutrophils (Mac1, 101207, clone M1/70; Biolegend, Amsterdam, The Netherlands). For NIMP (HM1039PE‐100, clone NIMP‐R14; Hycult Biotech, Uden, The Netherlands) staining, no amplification step was needed and an anti‐rat:peroxidase conjugate was used for the second layer. For the incubation step with the first antibody, we used 4% FCS/1× PBS + 1st antibody (Mac1, 1:500; NIMP, 1:100) + 1:5 Biotin Block solution (ABC kit) (Vector Laboratories). Incubation with the second antibody (1:300 anti‐rat:biotin conjugate or 1:100 anti‐rat:peroxidase conjugate) was in 4% FCS/2% normal mouse serum (Invitrogen, Carlsbad, CA, USA)/1× PBS. After this, the slides were washed and incubated in 1× PBS + 1:50 Biotin solution (ABC kit) (Vector Laboratories). An AEC kit (2% buffer/3% AEC/2% H_2_O_2_; Bio‐connect, Huissen, The Netherlands) in demineralized water was used to develop color and hematoxylin (Klinipath, Olen, Belgium) for nuclear counterstaining. Sections were mounted under glass using Faramount aqueous mounting medium (Agilent Technologies, Santa Clara, CA, USA).

Sections of paraffin‐embedded liver, cut at 4 μm, were stained with hematoxylin and eosin (H&E) after dewaxing in xylene and rehydrating with 90%, 70%, and 50% ethanol. Sections were mounted using Entellan (Merck, Branchberg, NJ, USA). Pictures were taken using a Nikon DMX1200 digital camera and ACT‐1 v2.63 software (Nikon, Tokyo, Japan). Immune cells were counted in six microscopic views (original magnification 200×) and were recorded as cells per square millimeter.

### Cell culture

BMDMs were isolated from the tibiae and femurs of male or female *Npc1* ^*nih*^ and *Wt* C57BL/6 mice. Cells were cultured for 8 days in RPMI‐1640 supplemented with 10% heat‐inactivated new‐born calf serum, penicillin (100 U/ml), streptomycin (100 μg/ml), and l‐glutamine (2 mm), supplemented with 20% L929‐conditioned medium (LCM) to generate BMDMs. After attachment, BMDMs were seeded at 350 000 cells per well in 24‐well plates. A complete overview of the experiment is depicted in supplementary material, Figure S1. At the end of the experiment, medium was collected and cells were lysed for mRNA expression or protein analysis. All *in vitro* data represent at least *n* = 3 (triplicates) for each experimental group.

### Nitric oxide measurement

Levels of nitric oxide (NO) were measured in medium using the Griess [0.1% naphthylethylenediamine dihydrochloride (Sigma‐Aldrich, St Louis, MO, USA); 1% sulfanilamide (Sigma‐Aldrich); 2.5% phosphoric acid (Merck)] test protocol. A standard curve was made using dilutions of NaNO_2_ in E2 (Sigma‐Aldrich) medium: 10, 8.75, 7.5, 6.25, 5, and 3.75 μl (E2 medium blank), and 50 μl of standard dilution and 45 μl of samples + 5 μl of 50 μm NaNO_2_ were pipetted into the wells of a 96‐well plate. Griess agent (50 μl) was added and Absorbance was measured at 545 nm.

### Tumor necrosis factor alpha (TNFα), interleukin 10 (IL‐10), estrogen receptor alpha (ERα) and estrogen receptor beta (ERβ), liver X receptor beta (LXRβ) enzyme linked immunosorbent assays (ELISA)

ELISA for mouse TNFα (17644800; Invitrogen) and IL‐10 (218449‐002; Invitrogen) were performed on culture medium of bone marrow‐derived macrophages according to the manufacturer’s instructions [sensitivity: 8 pg/ml (TNFα), 23 pg/ml (IL‐10); specificity: cross‐contamination from other species possible]. Protein levels of ERα (E‐EL‐M0476; Elabscience, Wuhan, PR China) and ERβ (E‐EL‐M0490; Elabscience) in cell lysates of bone marrow‐derived macrophages were measured by ELISA according to the manufacturer’s instructions (ERα sensitivity: 0.19 ng/ml and ERβ sensitivity: 0.19 ng/ml). No significant cross‐reactivity or interference between ERα and ERβ was observed. LXRβ (MOEB0190; Medical Supply Company, Dublin, Ireland) was measured in cell lysates of *Wt* bone marrow‐derived macrophages according to the manufacturer’s instructions.

### Statistical analysis

The differences between the experimental groups were analyzed with two‐way ANOVA followed by Tukey’s *post hoc* test using the IBM® SPSS Statistics program (Version 22.0.0.) or GraphPad Prism (Version 6.01; GraphPad Inc, San Diego, CA, USA). GraphPad Prism (Version 6.01) was used to calculate and display results as mean ± SEM (*p ≤ 0.05; **p ≤ 0.01). Pearson correlation coefficients (*r*) and respective *P* values were calculated to assess the statistical significance of correlations.

## Results

### Plasma 27‐hydroxycholesterol levels are oppositely associated with hepatic inflammatory indicators between female and male obese individuals

**Figure 1:**
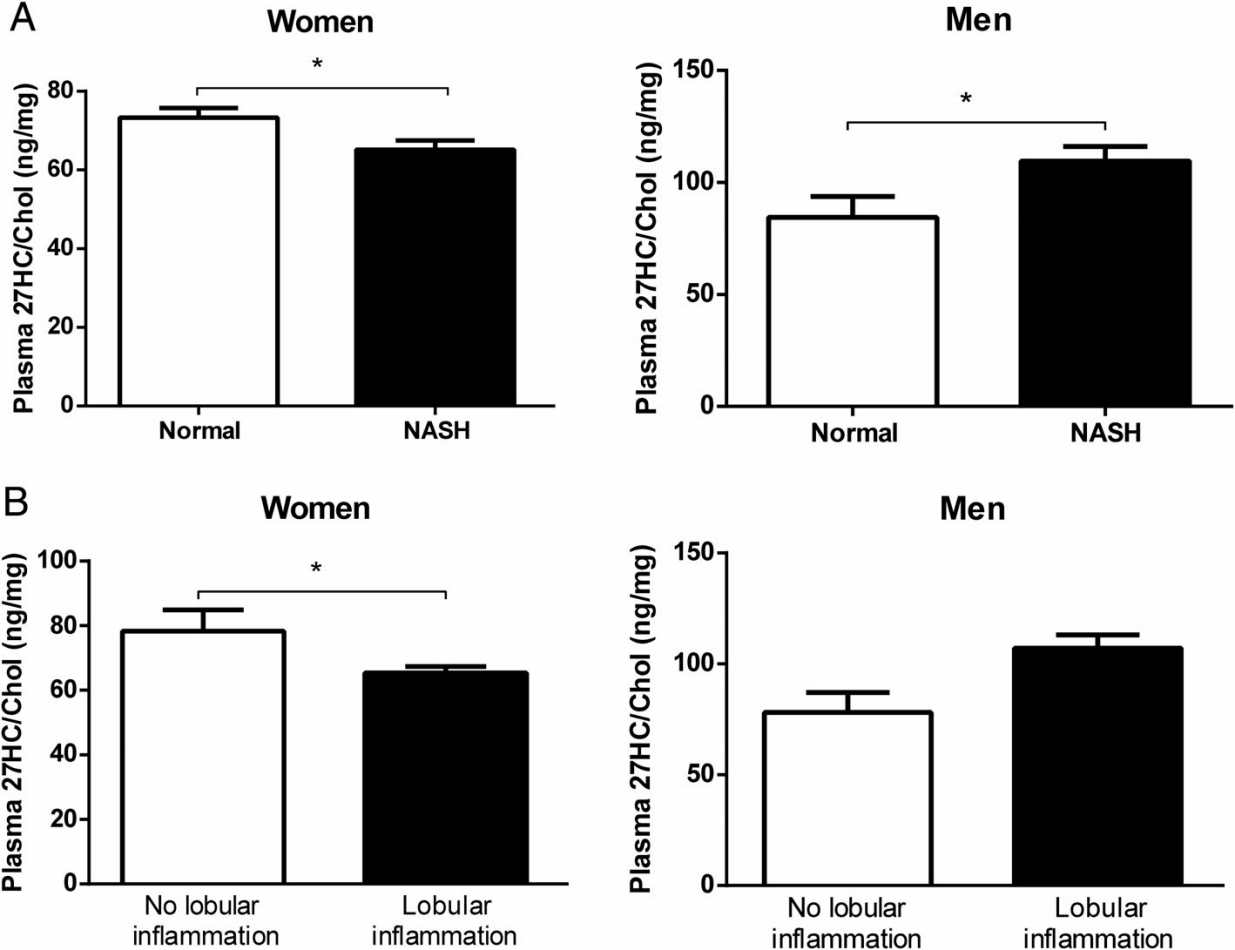
Relative plasma 27HC levels in female and male obese individuals in relation to hepatic inflammatory indicators. (A) Plasma 27HC levels in obese women and men categorized as ‘NASH’ or ‘Normal’ based on assessment of liver biopsies according to the criteria of Brunt. (B) Plasma 27HC levels in female and male obese individuals categorized according to the presence of hepatic lobular inflammation. *p ≤ 0.05 compared with obese individuals categorized as ‘Normal’ (A) or obese individuals without hepatic lobular inflammation (B) by use of two‐tailed unpaired *t‐*test. Panels A and B show *n* = 34 (21 female and 13 male individuals). All error bars are SEM.

To explore whether sex is associated with the inflammatory effect of 27HC, plasma 27HC levels were measured in an obese population and correlated with different hepatic inflammatory indicators. Clinical characteristics of the population are presented in supplementary material, Table S3. In the ‘Maastricht cohort’ (*n* = 43), plasma 27HC levels were significantly lower in female NASH individuals (categorized by criteria of Brunt) than in no‐NASH obese females (Figure 1A and supplementary material, Figure S2A, left panel). Conversely, in male NASH individuals, plasma 27HC levels were increased compared with no‐NASH obese males (Figure 1A and supplementary material, Figure S2A, right panel). Similarly, when plasma 27HC levels were specifically compared with hepatic lobular inflammation in the same obese population, significantly lower plasma 27HC levels were observed in obese females suffering from hepatic lobular inflammation (Figure 1B and supplementary material, Figure S2B, left panel), while an increasing trend was found for plasma 27HC levels in obese males (Figure 1B and supplementary material, Figure S2B, right panel). These results show that plasma 27HC levels oppositely correlate with hepatic inflammatory indicators in female and male obese individuals, confirming the sex‐opposed inflammatory association with 27HC that was previously observed in hyperlipidemic models [20, 21, 22].

### Hepatic 27HC levels oppositely correlate with hepatic inflammation between female and male *Npc1* ^*nih*^ mice

To validate the sex‐opposed inflammatory effect of 27HC *in vivo*, hepatic inflammation and hepatic 27HC levels were assessed in 7‐week‐old female and male Niemann–Pick type C1 disease (NPC1) mice (*Npc1* ^*nih*^ mice). Cryosections of liver were stained for the inflammatory markers Mac1 (infiltrated macrophages and neutrophils) and NIMP (neutrophils), and subsequently correlated with hepatic 27HC levels. Hepatic 27HC levels inversely correlated with both hepatic infiltrated macrophages and neutrophils (*r* = −0.8605; Table 1 and supplementary material, Figure S3A,B) and hepatic neutrophils (*r* = −0.9669; Table 1 and supplementary material, Figure S3A,B) in female *Npc1* ^*nih*^ mice. In contrast, male *Npc1* ^*nih*^ mice showed a positive correlation between hepatic 27HC levels and hepatic infiltrated macrophages and neutrophils (*r* = 0.9595; Table 1 and supplementary material, Figure S3A,B) and hepatic neutrophils (*r* = 0.88; Table 1 and supplementary material, Figure S3A,B). Furthermore, no other significant differences were observed between female and male *Npc1* ^*nih*^ mice (supplementary material, Table S4). As such, our data show that hepatic 27HC levels have sex‐opposed hepatic inflammatory associations, validating *Npc1* ^*nih*^ mice as a model to investigate the sex‐opposed inflammatory effects of 27HC.

**Table 1.** Correlation analysis between hepatic 27‐hydroxycholesterol levels and hepatic inflammation in 7‐week‐old female and male *Npc1* ^*nih*^ mice.

	Hepatic 27HC/Chol levels (ng/mg)		
	Pearson *r*	*R* ^2^	*P* value
Female			
Hepatic infiltrated macrophages and neutrophils (No of positive cells/mm^2^)	−0.8605	0.7404	0.13
Hepatic neutrophils (No of positive cells/mm^2^)	−0.9669	0.9349	0.03*
Male			
Hepatic infiltrated macrophages and neutrophils (No of positive cells/mm^2^)	0.9595	0.9206	0.04*
Hepatic neutrophils (No of positive cells/mm^2^)	0.8861	0.7851	0.11

* *p* ≤ 0.05 by means of Pearson correlation. *n* = 4 per experimental group.

### Administration of 27HC in *Npc1* ^*nih*^ mice results in opposite hepatic inflammatory responses between sexes

**Figure 2:**
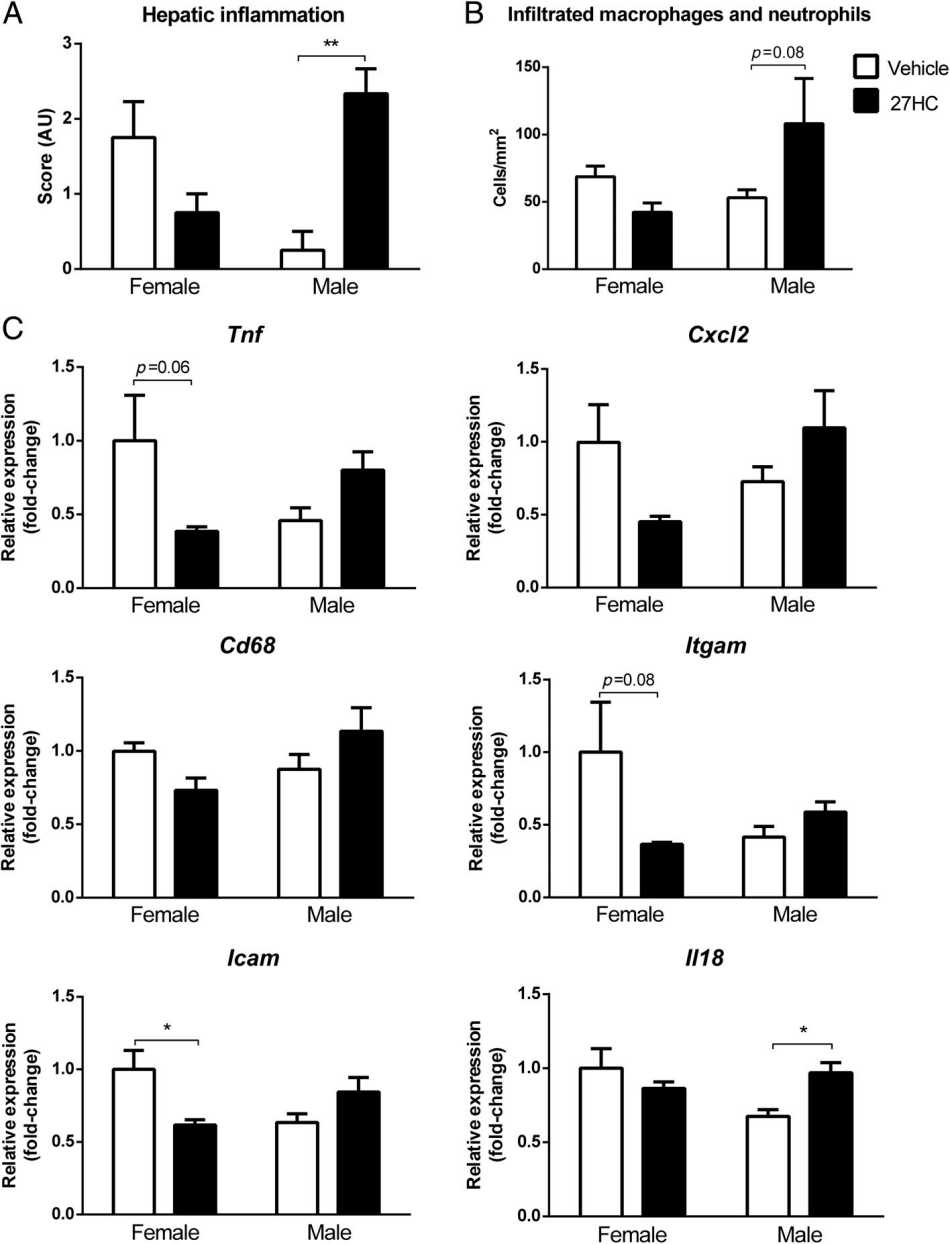
Hepatic inflammatory parameters of female and male *Npc1* ^*nih*^ mice after 27HC administration. (A, B) Quantification of hematoxylin and eosin (H&E) staining and Mac1 immunostaining of livers from female and male *Npc1* ^*nih*^ mice treated with or without 27HC. (C) Hepatic gene expression analysis of *Tnf*, *Cxcl2*, *Cd68*, *Itgam*, *Icam1*, and *Il18* of female and male *Npc1* ^*nih*^ mice treated with or without 27HC. *p ≤ 0.05 and **p ≤ 0.01 by use of two‐way ANOVA with Tukey’s *post hoc* correction. *n* = 4 or 5 mice per experimental group. All error bars are SEM.

To determine whether 27HC induces the sex‐opposed effects on hepatic inflammation, female and male *Npc1* ^*nih*^ mice were given daily subcutaneous injections of 27HC or vehicle for 21 weeks. Hepatic and bile 27HC levels increased in both female and male *Npc1* ^*nih*^ mice upon 27HC administration, confirming successful administration of 27HC (supplementary material, Figure S4). In line with our hypothesis, administration of 27HC in female *Npc1* ^*nih*^ mice reduced hepatic inflammation, as observed by H&E staining (Figure 2A and supplementary material, Figure S5) and decreased numbers of infiltrated macrophages and neutrophils (Figure 2B) in comparison to non‐treated animals. In contrast, administration of 27HC in male *Npc1* ^*nih*^ mice resulted in a pro‐inflammatory response compared with vehicle‐treated male *Npc1* ^*nih*^ mice (Figure 2A,B). These histological observations were further substantiated by hepatic gene expression levels for the inflammatory markers tumor necrosis factor alpha (*Tnf*), chemokine (C‐X‐C motif) ligand 2 (*Cxcl2*), cluster of differentiation 68 (*Cd68*), integrin alpha M (*Itgam*), intercellular adhesion molecule 1 (*Icam1*), and interleukin 18 (*Il18*) (Figure 2C), showing tendencies towards decreased or increased inflammation in female or male *Npc1* ^*nih*^ mice, respectively. Moreover, the interaction term provided by two‐way ANOVA analysis was significant among all hepatic inflammatory analyses (Table 2), indicating that the hepatic inflammatory effect of 27HC is influenced by sex.

**Table 2.** Statistics of sex‐opposed hepatic inflammatory effects of 27HC in *Npc1* ^*nih*^ mice.

	*P* value (sex*27HC treatment interaction term)
Histology	
Hepatic inflammation (H&E)	0.001**
Infiltrated macrophages and neutrophils	0.0181*
Gene expression	
*Cd68*	0.0351*
*Tnf*	0.0066**
*Cxcl2*	0.04*
*Il18*	0.0142*
*Icam1*	0.0051**
*Itgam*	0.0256*

*p ≤ 0.05 and **p ≤ 0.01 by two‐way ANOVA. *n* = 4 or 5 mice per experimental group.

### Only in the presence of E2 are the inflammatory effects of 27HC sex‐dependent in murine bone marrow‐derived macrophages

**Figure 3:**
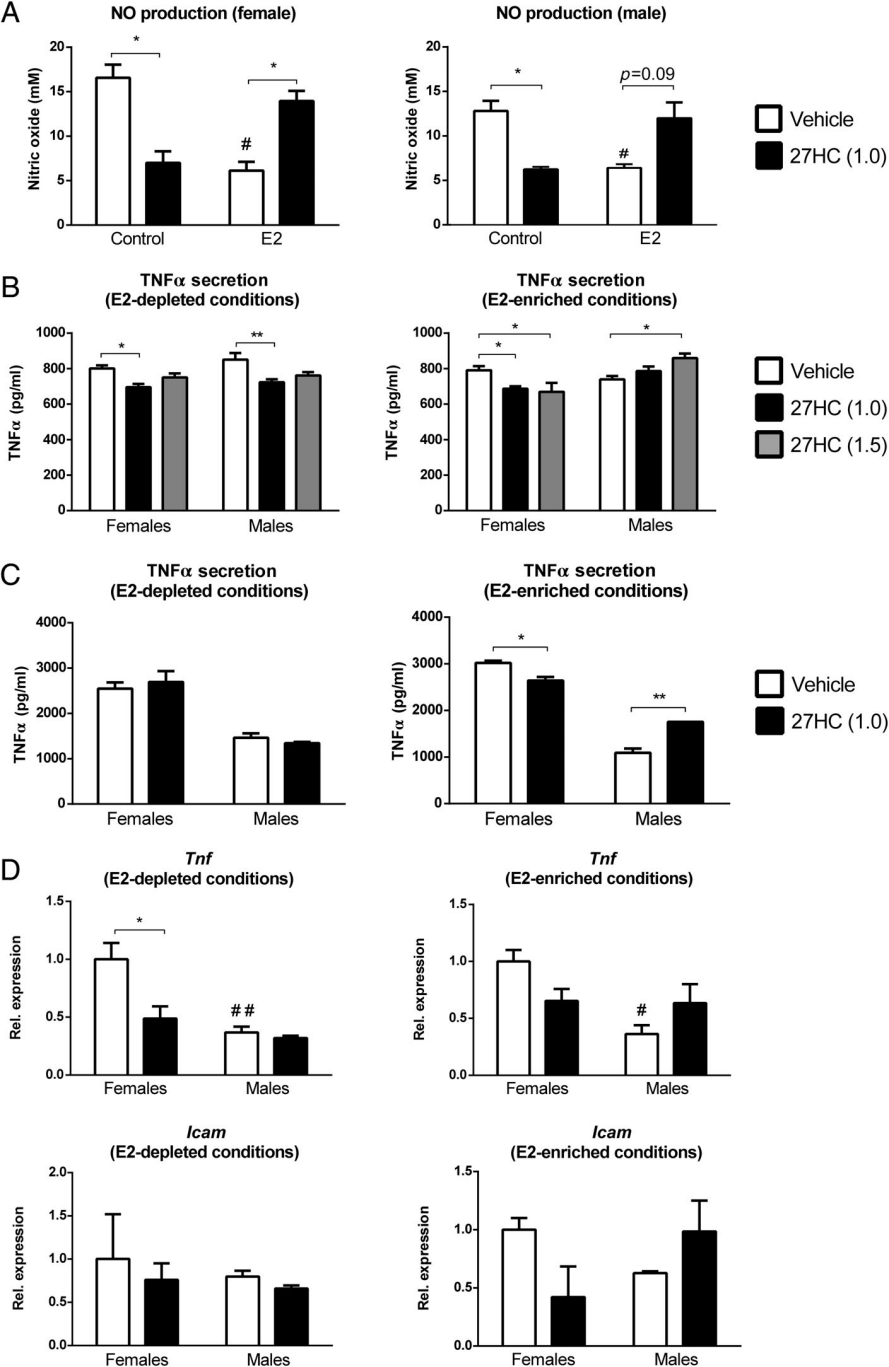
Inflammatory indicators of female and male‐derived *Wt* bone marrow‐derived macrophages cultured in E2‐enriched/‐depleted medium treated with 27HC. (A) Nitric oxide (NO) production in female (left panel) and male (right panel) wild‐type BMDMs. *p ≤ 0.05 compared with vehicle‐treated BMDMs by use of two‐way ANOVA with Tukey’s *post hoc* correction. ^#^ *p* ≤ 0.05 compared with E2‐depleted (‘Control’) BMDMs treated with vehicle. (B) TNFα protein levels in female and male *Wt* BMDMs. *p ≤ 0.05 and **p ≤ 0.01 compared with vehicle‐treated female‐ or male‐derived BMDMs cultured under E2‐depleted conditions by use of two‐way ANOVA with Tukey’s *post hoc* correction (left panel). *p ≤ 0.05 is compared with vehicle‐treated female‐ or male‐derived BMDMs cultured under E2‐enriched conditions (right panel). Results are shown as the average of three independent experiments. All error bars are SEM. (C) TNFα protein levels in female and male *Npc1* ^*nih*^ BMDMs. *p ≤ 0.05 and **p ≤ 0.01 compared with vehicle‐treated female‐ or male‐derived BMDMs cultured under E2‐enriched conditions by use of two‐way ANOVA with Tukey’s *post hoc* correction (right panel). Results are shown as the average of one independent experiment. All error bars are SEM. (D) Gene expression analysis of *Tnf* and *Icam1* in female and male *Npc1* ^*nih*^ BMDMs. *p ≤ 0.05 compared with vehicle‐treated female‐ or male‐derived BMDMs cultured under E2‐enriched conditions by use of two‐way ANOVA with Tukey’s *post hoc* correction. ^#^ *p* ≤ 0.05 and ^##^ *p* ≤ 0.01 compared with vehicle‐treated female BMDMs by use of two‐way ANOVA with Tukey’s *post hoc* correction. Results are shown as the average of one experiment with three technical replicates. All error bars are SEM.

As 27HC has been identified as a selective estrogen receptor modulator (SERM) [14] and estrogen is known to influence inflammatory processes [29], we first assessed the effect of 27HC in murine BMDMs cultured in E2‐depleted and ‐enriched conditions by measuring nitric oxide (NO), a key mediator of inflammation. Vehicle‐treated BMDMs cultured in E2‐enriched medium showed reduced production of NO compared with vehicle‐treated BMDMs cultured in E2‐depleted (‘Control’) medium, confirming the modulatory role of E2 in NO production (Figure 3A,B). Strikingly, treating BMDMs cultured in E2‐enriched medium with 27HC resulted in increased NO production, which was opposite in BMDMs cultured in E2‐depleted medium, where treatment with 27HC reduced NO production (Figure 3A,B). These results confirm that 27HC modifies the estrogen‐mediated inhibition of NO production, and therefore inflammation.

Next, we investigated whether the sex‐opposed inflammatory effects of 27HC are E2‐dependent. For this purpose, *Wt* BMDMs derived from female or male mice were cultured in E2‐depleted or E2‐enriched medium and treated with 27HC (1.0 or 1.5 μm) or vehicle for 24 h. Under E2‐depleted conditions, 27HC treatment reduced TNFα levels in female‐ and male‐derived BMDMs (Figure 3B, left panel). In contrast, under E2‐enriched conditions, 27HC treatment only reduced TNFα levels in females, but not in males (Figure 3B, right panel). Moreover, upon incubation with 1.5 μm 27HC, TNFα levels were significantly increased in males (Figure 3B, right panel). These observations were confirmed at gene expression level, showing similar trends for *Tnf*, *Icam1*, and *Il1b* expression (supplementary material, Figure S6A). Sex, however, did not influence IL‐10 levels after 27HC treatment (supplementary material, Figure S6B). Additionally, to confirm whether these inflammatory effects were more pronounced in an extreme model of cholesterol‐induced inflammation, female‐ and male‐derived *Npc1* ^*nih*^ BMDMs were exposed to conditions similar to those of *Wt* BMDMs. As expected, only under E2‐enriched conditions did the sex‐opposed inflammatory effects of 27HC become apparent, demonstrating reduced TNFα levels in females and increased TNFα levels in males (Figure 3C and Table 3). This observation was also further confirmed at gene expression level for *Tnf* and *Icam1* (Figure 3D and Table 3). Together, these findings indicate that the sex‐opposed inflammatory effects of 27HC are E2‐dependent and imply that the downstream E2 signaling is different in females and males.

**Table 3.** Statistics of sex‐opposed inflammatory effects of 27HC in *Npc1* ^*nih*^ BMDMs.

	*P* value (sex*27HC treatment interaction term)
TNFα secretion (protein)	
E2‐depleted	0.3805
E2‐enriched	0.0014**
*Tnf* (mRNA expression)	
E2‐depleted	0.0360*
E2‐enriched	0.0468*
*Icam1* (mRNA expression)	
E2‐depleted	0.8107
E2‐enriched	0.0451*

*p ≤ 0.05 and **p ≤ 0.01 by two‐way ANOVA. *n* = 3 per experimental group.

### Female BMDMs show higher ERα expression compared with male BMDMs

**Figure 4:**
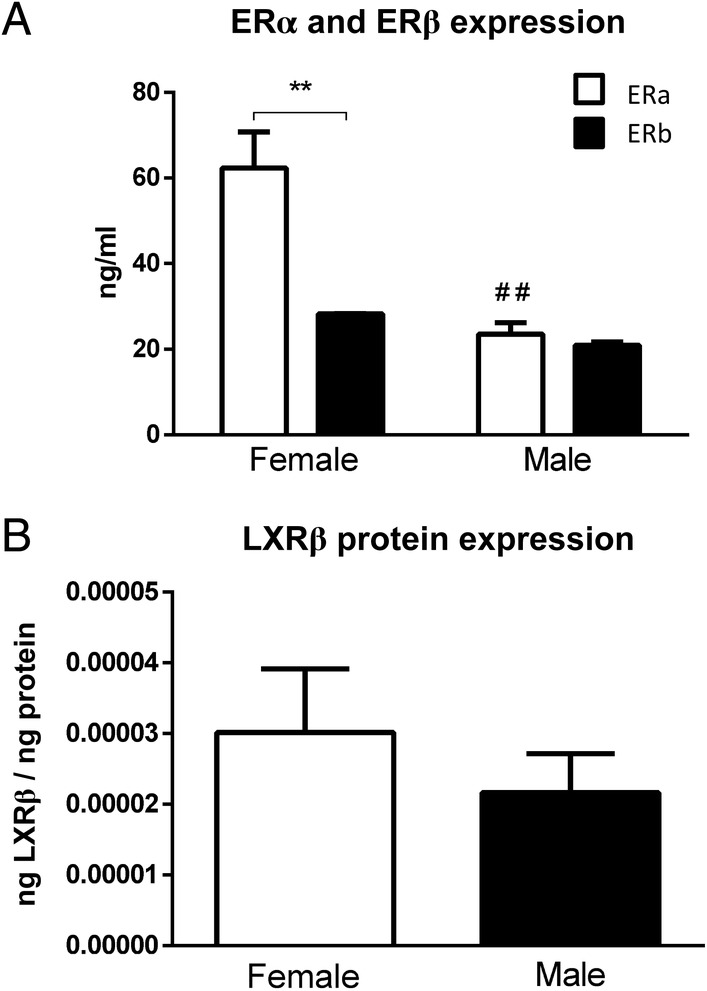
Protein expression of ERα, ERβ, and LXRβ in female and male *Wt* bone marrow‐derived macrophages. (A) ERα and ERβ and (B) LXRβ expression in female and male BMDMs. **p ≤ 0.01 compared with female ERβ expression and ^##^ *p* ≤ 0.01 compared with female ERα expression by use of two‐way ANOVA with Tukey’s *post hoc* correction. Results are shown as the average of three independent experiments. All error bars are SEM.

To provide insight into the underlying mechanism that explains the E2‐driven opposite inflammatory effect of 27HC on females and males, we determined protein levels of estrogen receptors alpha and beta (ERα and ERβ) and liver X receptor beta (LXRβ) in untreated *Wt* BMDMs derived from female and male mice (Figure 4A,B). While ERβ and LXRβ levels were similar between females and males, ERα levels showed almost three‐fold higher protein expression in female compared with male BMDMs (Figure 4A,B). Moreover, in females, ERα expression was two‐fold higher than ERβ expression, while in males ERα and ERβ showed almost equal protein expression (Figure 4A). Considering the modulating effect of these estrogen receptor subtypes on inflammatory responses, our findings suggest that the sex‐opposed inflammatory effect of 27HC might be related to different ERα expression between female and male macrophages.

## Discussion

Despite the increased awareness of differences in the inflammatory response between men and women, only limited research has focused on the biological factors underlying these sex‐opposed effects. Here, we identify 27HC as a biological factor exerting sex‐opposed effects on inflammation and show that these sex‐opposed effects are mediated via differences in estrogen signaling. Hence, the individual’s sex needs to be taken into account when 27HC is employed as a therapeutic tool as well as in macrophage estrogen research in general.

An increasing amount of research is pointing towards the therapeutic potential of bile acids (and their precursors) to modulate the inflammatory response in metabolic diseases such as cardiovascular diseases [30], diabetes [31], NASH [32], and even in pulmonary diseases [33, 34]. With regard to this evolving trend, we demonstrate in the current study that sex plays a key role in the inflammatory effect of the bile acid precursor 27HC, which likely explains the previous contradictive results in atherosclerosis [22] and NASH [20, 21]. Our findings suggest that bile acids and their precursors potentially have opposite inflammatory effects in females and males, warranting caution on the use of bile acids as a therapeutic tool in the clinic without conducting sex‐specific clinical trials. Indeed, in the past, sex bias has been identified in the search for new therapeutic drugs in both preclinical animal studies and clinical human studies that included more male than female individuals [35, 36]. In the cardiovascular field, this phenomenon has led to differences in the clinical outcome of males versus females in relation to key drugs such as beta‐blockers, angiotensin‐converting enzyme inhibitors, diuretics, and anti‐arrhythmics [36]. As, to our knowledge, this is the first study to describe sex‐related differences in the context of bile acids, future research is necessary to validate our observation. So far, gender‐specific health care is only present in very limited quantities and epidemiological research proving sex differences [37, 38] are only recently finding their way to larger scientific audiences, which is exemplified by funding agencies specifically endorsing research investigating female/male differences [39, 40]. Our findings therefore add fuel to the increasing concept of men and women as two different entities whose health and disease condition should be approached from a different perspective.

While a chronic low‐grade inflammatory state is as a key factor contributing to the prevalence of obesity‐associated disorders [6, 7, 41], only recently has it become clear that there are differences between the sexes concerning this obesity‐associated inflammatory response. Plasma adiponectin concentrations were demonstrated to decrease more dramatically in obese male than in female patients [42], providing evidence for a more detrimental inflammatory state in obese male compared with obese female patients. Additionally, whereas male high‐fat diet‐induced obese mice showed profound adipose tissue macrophage accumulation [43] and infiltration [44], female obese mice were protected from this adipose tissue inflammatory response [43, 44]. Singer *et al* suggested that the absence of adipose tissue inflammation in female high‐fat diet‐induced obese mice is likely due to the formation of CD11c^−^ (M2‐like) macrophages, while matched male mice show expansion of CD11c^+^ (M1‐like) macrophages in adipose tissue [45]. These findings suggest a key role for macrophages in mediating opposite inflammatory responses between females and males. In line with this view, we observed here that the cholesterol derivative 27HC exerts anti‐inflammatory effects in the livers of females and pro‐inflammatory effects in the livers of males and that we could replicate these findings in primary macrophages as well as validate them in a cohort of obese individuals. The sex‐dependent inflammatory influence of 27HC on macrophages has been confirmed in other experiments showing that 27HC exerts pro‐inflammatory effects on male‐derived macrophages [46], whereas anti‐inflammatory effects are observed in female‐derived macrophages [20]. Therefore, future studies focusing on the inflammatory effect of 27HC in macrophages have to consider sex as a potential variable influencing the outcome of their studies.

The observation that the presence of estrogen is essential to observe the sex‐opposed inflammatory effects of 27HC in BMDMs indicates a key role for estrogen in the 27HC‐induced effect on inflammation. This observation is in line with previous findings of Umetani *et al*, who linked 27HC to estrogen by identifying 27HC as the first endogenous selective estrogen receptor modulator (SERM) [14]. Estrogens are known to dampen, reduce, and inhibit inflammatory responses by various mechanisms, including via activation of ER dimers that repress transcription of, for instance, iNOS [47]. Indeed, we found that estrogen reduces NO production and, additionally, that administration of 27HC in the absence of E2 resulted in a similar decrease in NO production, suggesting a similar effect of E2 and 27HC on ER dimers. Simultaneous administration of E2 and 27HC, on the other hand, causes a conformational change in ER dimers [47], blocking their repressive effect on transcription and explaining the increased NO production. These observations therefore confirm that 27HC is a SERM. Additionally, besides ER dimers, estrogens also influence inflammation via repressing the transcription factor NF‐κB [29], a master regulator of inflammation [48]. Indeed, we found that downstream targets of NF‐κB, TNFα, ICAM, and IL‐1β, decreased in female‐derived, but not male‐derived, BMDMs cultured in E2‐enriched medium that were treated with 27HC. As production of the inflammatory mediator NO (which is not NF‐κB‐mediated) [49] was not differentially influenced by 27HC among the sexes, our observations provide evidence that the sex‐opposed inflammatory effect of 27HC is likely related to NF‐κB‐mediated mechanisms.

Additionally, our findings further support that the sex‐opposed inflammatory effects of 27HC are at least partly mediated via differences in downstream estrogen signaling of macrophages. This observation raises the question of how downstream estrogen signaling in macrophages is different in females and males. While estrogen exerts anti‐inflammatory effects in both females and males [50], a potential answer to this exciting question might be related to sex differences in the distribution of the different ERs, ERα and ERβ. Indeed, while ERβ expression was similar, we observed a substantial increased expression of ERα in female compared with male BMDMs, suggesting that females show more pronounced ERα‐mediated macrophage responses that underlie the observed anti‐inflammatory effect of 27HC in females. In line with this observation, ERα expression in macrophages has been linked to anti‐inflammatory responses in skin repair [51] and infectious immunity [52]. Moreover, while ERα and ERβ share a high degree of homology, their target genes differ substantially, indicating that the binding of ligands (such as 27HC) to different ER subtypes impacts the fate of the inflammatory signaling [53]. For these reasons, future research should further investigate whether differences in the distribution of ERα and ERβ determine the inflammatory fate of 27HC. The different ERα expression levels between females and males also highlight sex as an essential factor to be considered when investigating macrophage estrogen signaling, irrespective of 27HC levels. As such, for future research, it is indispensable to determine whether the macrophages under investigation are derived from female or male sources.

As cholesterol levels increase during obesity in men and women [54] and 27HC levels are tightly correlated with cholesterol levels, our findings suggest that increased cholesterol levels will result in a more detrimental inflammatory response in males than in females. Indeed, previous reports have described that premenopausal women are better protected from metaflammatory insults compared with male individuals [55]. Additionally, the premenopausal inflammatory resilience in women reverses after menopause [56], suggesting that estrogen plays a key role in mediating the metaflammatory response. Nevertheless, estrogen administration in postmenopausal women resulted in a paradoxical increased risk of inflammation and cardiovascular disease [57, 58], suggesting that other biological factors might be involved in the inflammatory response in females. Considering our findings that 27HC is affecting inflammation via perturbing estrogen signaling and the previous observation of increased LDL cholesterol (and concomitantly 27HC) levels after menopause [59], an interesting line for future research would be to explore whether the inflammatory effect of 27HC is different in pre‐ versus post‐menopausal women.

Overall, we have shown here that 27HC exerts sex‐opposed effects on cholesterol‐induced inflammatory responses that are potentially mediated via sex‐related expression differences of ERα. Future studies should investigate how these findings have implications for gender‐based therapeutic strategies against inflammation.

## Author contributions statement

AB, TH, YO, MJ, DL, JT, JP, AR, and RSS conceived and designed the study. AB, TH, YO, and MJ were involved in acquisition of data. AB, TH, JP, AR, and RSS performed the (statistical) analysis and interpretation of data. AB, TH, YO, and RSS drafted the manuscript. All the authors were involved in revising the paper critically and gave final approval of the version to be submitted.

## Supporting information


**Figure S1.** *In vitro* experimental set‐up
**Figure S2.** Plasma absolute 27HC levels in female and male obese individuals in relation to hepatic inflammatory indicators
**Figure S3.** Hepatic inflammation in female and male *Npc1* ^*nih*^ mice
**Figure S4.** Bile acids in female and male *Npc1* ^*nih*^ mice
**Figure S5.** H&E‐stained liver tissue from female and male *Npc1* ^*nih*^ mice
**Figure S6.** Inflammatory profiling of female and male‐derived *Wt* bone marrow‐derived macrophages cultured in E2‐enriched/‐depleted medium treated with 27HC

**Table S1.** Product information
**Table S2.** Primer sequences for RT‐qPCR
**Table S3.** Population characteristics of the Maastricht cohort
**Table S4.** Characteristics of 7‐week‐old *Npc1* ^*nih*^ mice
