# Supplementary material for: Sex‐opposed inflammatory effects of 27‐hydroxycholesterol are mediated via differences in estrogen signaling
Source: J Pathol. 2020 Jul 7;251(4):429–39. doi: 10.1002/path.5477 (PMC7497011; doi:10.1002/path.5477)
Supplement: Supplementary file 1 — Figure S1. In vitro experimental set‐up Figure S2. Plasma absolute 27HC levels in female and male obese individuals in relation to hepatic inflammatory indicators Figure S3. Hepatic inflammation in female and male Npc1 nih mice Figure S4. Bile acids in female and male Npc1 nih mice Figure S5. H&E‐stained liver tissue from female and male Npc1 nih mice Figure S6. Inflammatory profiling of female and male‐derived Wt bone marrow‐derived macrophages cultured in E2‐enriched/‐depleted medium treated with 27HC [file PATH-251-429-s001.docx]

**Sex-opposed inflammatory effects of 27-hydroxycholesterol are mediated via differences in estrogen signaling**

T Houben, AV Bitorina *et al. J Pathol* DOI: 10.1002/path.5477


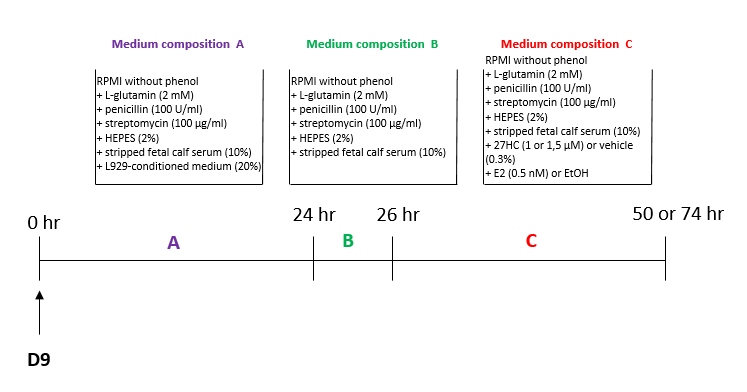


**Figure S1. *In vitro* experimental set-up.** After 9 days culture in regular RPMI medium (see Materials and methods section), BMDMs were incubated in medium A to get rid of estrogen in the RPMI medium and FCS. Next, BMDMs were cultured for 2 h without LCM medium (medium B) followed by 24 or 48 h incubation with 27HC (1 µm) or vehicle (2-hydroxypropyl-β-cyclodextrin; 0.3%) in combination with E2 (0.5 nm) or EtOH. HEPES, (4-(2-hydroxyethyl)-1-piperazineethanesulfonic acid); 27HC, 27-hydroxycholesterol; E2, 17β-estradiol, EtOH, ethanol.

**A**

**B**

**Figure S2. Plasma absolute 27HC levels in female and male obese individuals in relation to hepatic inflammatory indicators.** (A) Plasma 27HC levels in obese women and men categorized as ‘NASH’ or ‘Normal’ based on assessment of liver biopsies according to the criteria of Brunt. (B) Plasma 27HC levels in female and male obese individuals categorized according to the presence of hepatic lobular inflammation. **p*≤ 0.05 compared with obese individuals categorized as ‘Normal’ (A) or obese individuals without hepatic lobular inflammation (B) by use of two-tailed unpaired *t-*test. Panels A and B show *n*= 34 (21 female and 13 male individuals). All error bars are SEM.

**
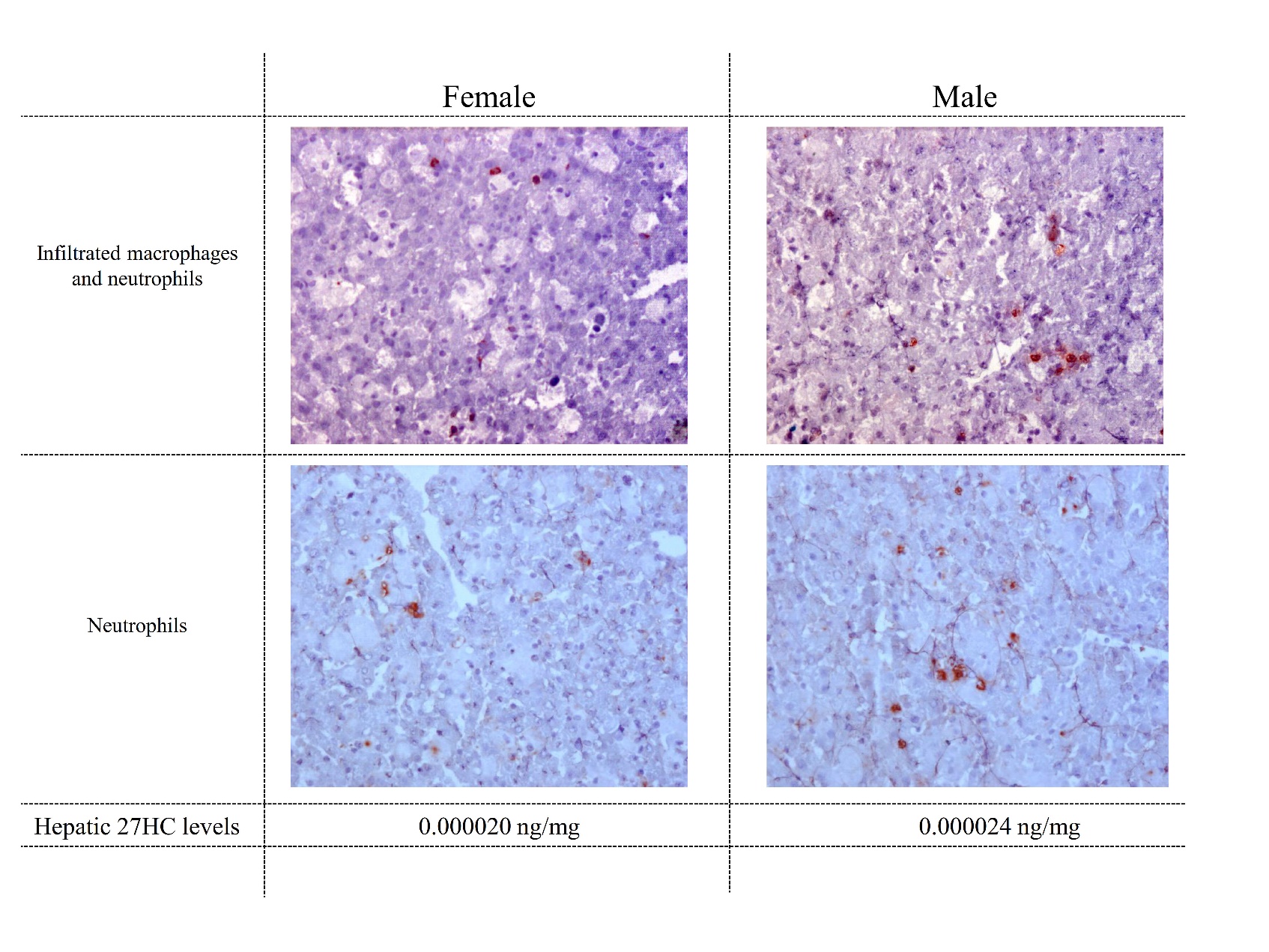

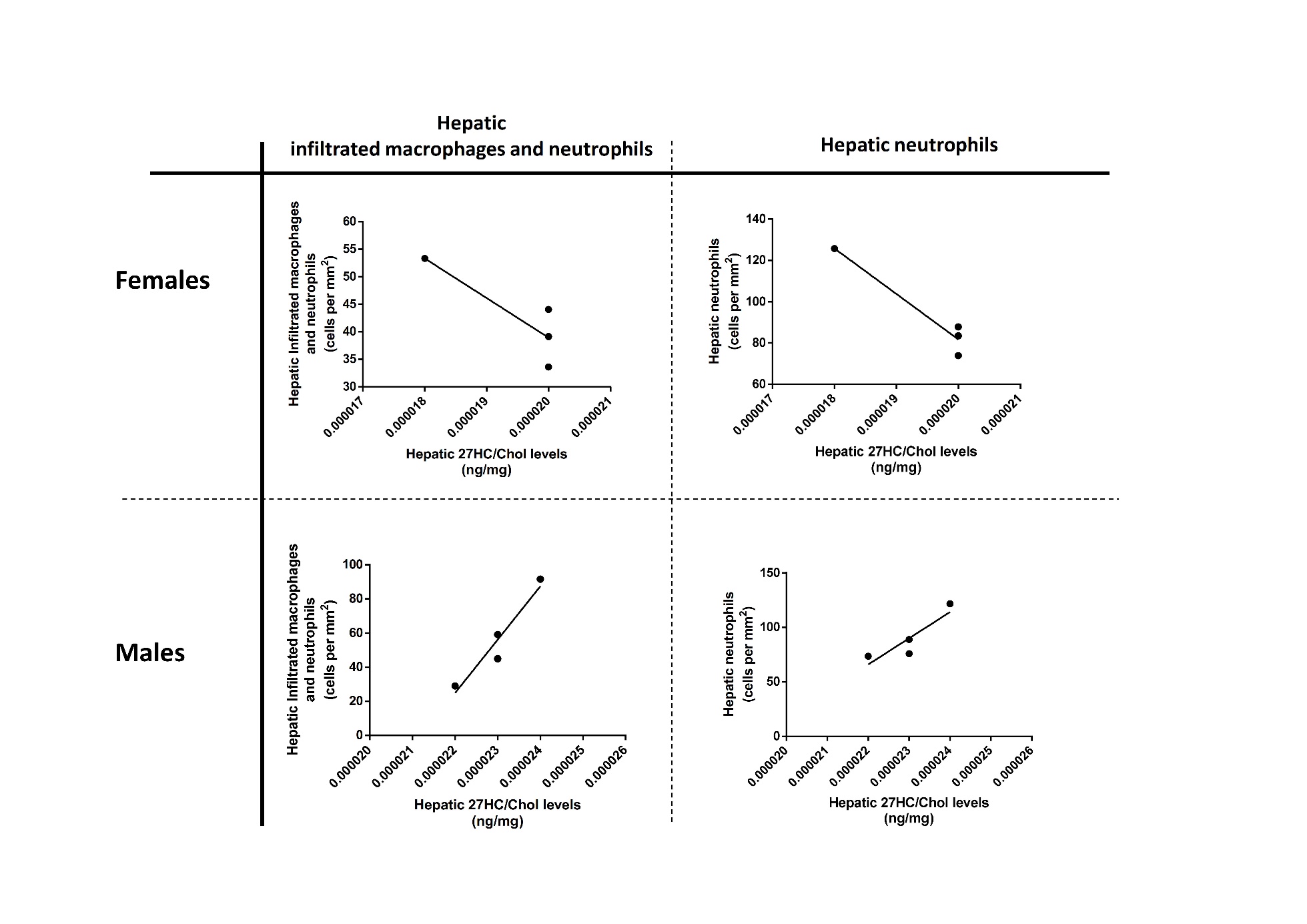
**

**B**

**A**

**Figure S3. Hepatic inflammation in female and male *Npc1^nih^* mice**. (A) Correlation analysis of hepatic 27HC levels with immunostaining quantification for hepatic infiltrated macrophages and neutrophils (Mac1) and hepatic neutrophils (NIMP staining) in 7-week-old female and male *Npc1^nih^* mice. *n* = 4 mice per experimental group. (B) Representative images (200× magnification) of a female and a male *Npc1^nih^* mouse with respective hepatic 27HC levels.

Vehicle

27HC (40 mg/kg)

**Figure S4. Bile acids in female and male *Npc1^nih^* mice.** Hepatic and bile 27HC levels in 27HC-administered *Npc1^nih^* mice. *n* = 4 or 5 mice per experimental group. **p* ≤ 0.05 and *****p* ≤ 0.0001 compared with vehicle-treated female or male *Npc1^nih^* mice by use of two-way ANOVA with Tukey’s *post hoc* correction. ^##^*p* ≤ 0.01 compared with 27HC-treated female *Npc1^nih^* mice. All error bars are SEM.


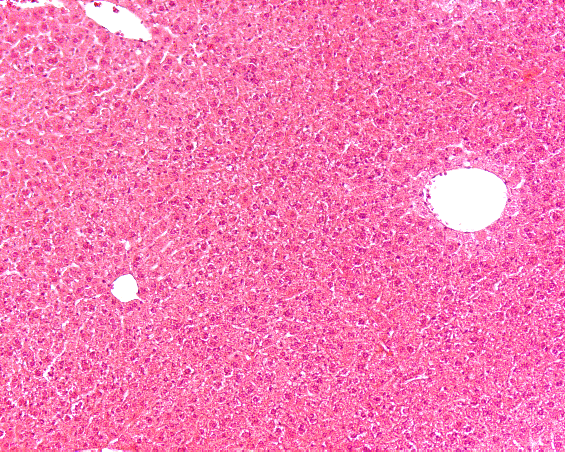

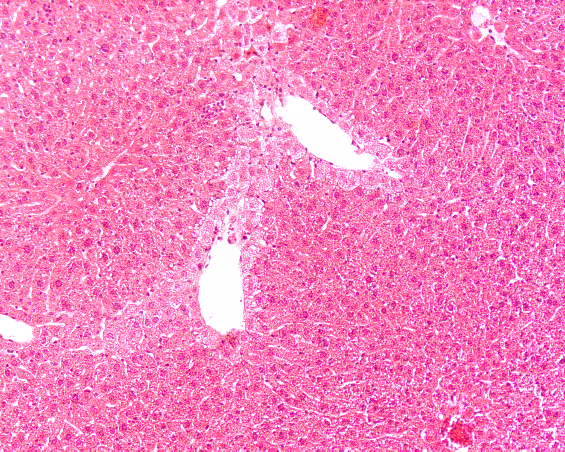

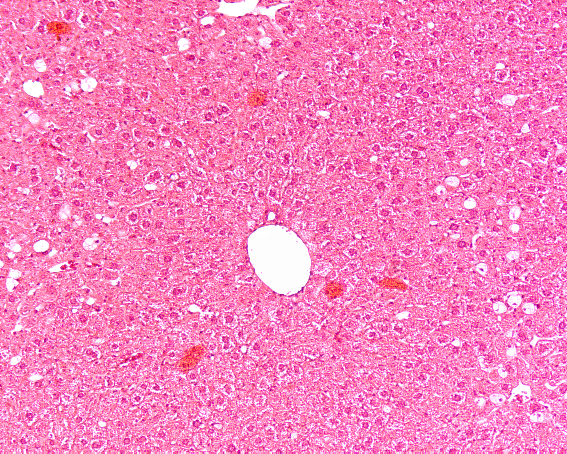

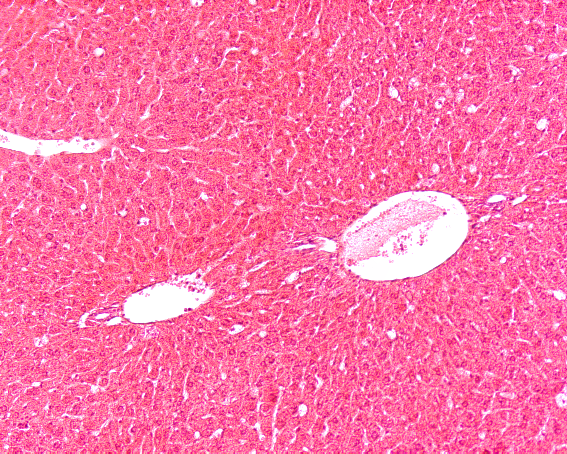


Female

Male

Vehicle

27HC

**Figure S5. H&E-stained liver tissue from female and male *Npc1^nih^* mice.** Representative images of H&E-stained liver tissue (100× magnification) of female and male *Npc1^nih^* mice that were treated with vehicle or 27HC for 12 weeks.

Vehicle

27HC (1.0 µM)

27HC (1.5 µM)

**A**

**B**

**Figure S6. Inflammatory profiling of female and male-derived *Wt* bone marrow-derived macrophages cultured in E2-enriched/-depleted medium treated with 27HC.** (A) Gene expression analysis of *Tnf*, *Icam1*, and *Il1b* in female and male *Wt* BMDMs. ^#^*p* ≤ 0.05 compared with vehicle-treated female BMDMs by use of two-way ANOVA with Tukey’s *post hoc* correction. (B) IL-10 protein levels in female and male *Wt* BMDMs. ***p* ≤ 0.01 compared with vehicle-treated female- or male-derived BMDMs cultured under E2-depleted conditions by use of two-way ANOVA with Tukey’s *post hoc* correction (left panel). ****p* ≤ 0.001 and *****p* ≤ 0.0001 are compared with vehicle-treated female or male BMDMs cultured under E2-enriched conditions (right panel). Results are shown as the average of two independent experiments. All error bars are SEM.
