# Supplementary material for: Sex‐opposed inflammatory effects of 27‐hydroxycholesterol are mediated via differences in estrogen signaling
Source: J Pathol. 2020 Jul 7;251(4):429–39. doi: 10.1002/path.5477 (PMC7497011; doi:10.1002/path.5477)
Supplement: Supplementary file 2 — Table S1. Product information Table S2. Primer sequences for RT‐qPCR Table S3. Population characteristics of the Maastricht cohort Table S4. Characteristics of 7‐week‐old Npc1 nih mice [file PATH-251-429-s002.docx]

**Sex-opposed inflammatory effects of 27-hydroxycholesterol are mediated via differences in estrogen signaling**

T Houben, AV Bitorina *et al. J Pathol* DOI: 10.1002/path.5477

**Table S1.** Product information

| **Product** | **Company** | **Catalog number** |
| --- | --- | --- |
| 27-Hydroxycholesterol | Obtained via Professor Plat |  |
| 2-Hydroxypropyl-beta-cyclodextrin | Sigma-Aldrich (St Louis, MO, USA) | H107 |
| Formaldehyde | Sigma-Aldrich | P6148-500g |
| Chloroform | Merck (Kenilworth, NJ, USA) | 1.02445.1000 |
| Isopropanol | Sigma-Aldrich | 33539-2.5L |
| Ethanol | Sigma-Aldrich | 32221-2.5L |
| DEPC sterile H_2_O | Sigma-Aldrich | D-5758 |
| iScript cDNA synthesis | Bio-Rad Inc (Hercules, CA, USA) | 170-8891 |
| Acetone | VWR (Radnor, PA, USA) | 20066.296 |
| H_2_O_2_ | Merck | 1.07210.1000 |
| Fetal calf serum | Bodinco (Alkmaar, The Netherlands) | BDS-12251 |
| ABC kit | Vector Laboratories (Burlingame, CA, USA) | PK-6100 |
| Mac1 antibody; rat-anti-mouse | Biolegend (Amsterdam, The Netherlands) | 101207 |
| Rat anti-mouse Ly6-C, NIMP | Hycult Biotech (Uden, The Netherlands) | HM1039PE-100 |
| Anti-rat-PO secondary antibody | Agilent Technologies (Santa Clara, CA, USA) | P0450 |
| Anti-rat-BIO secondary antibody | Southern Biotech (Birmingham, AL, USA) | 6180.08 |
| Normal mouse serum | Invitrogen (Carlsbad, CA, USA) | 31881 |
| AEC kit | Bio-connect (Huissen, The Netherlands) | A85SK-4200.S1 |
| Hematoxylin | Klinipath (Olen, Belgium) | 4085.9002 |
| Faramount aqueous mounting medium | Agilent Technologies | S302580 |
| Entallan | Merck | 1.07961.1000 |
| RPMI-1640 | GIBCO Invitrogen (Carlsbad, CA, USA) | 61870-010 |
| Penicillin and streptomycin | GIBCO Invitrogen | 15140-148 |
| l-Glutamine | GIBCO Invitrogen | A2916801 |
| 24-Well plates | Thermo Fisher (Waltham, MA, USA) | 662102 |
| Stripped estrogen serum | GIBCO Invitrogen | A33821-01 |
| Estradiol | Sigma-Aldrich | E2758-250MG |
| Naphthylethylenediamine dihydrochloride | Sigma-Aldrich | N9125 |
| Sulfanilamide | Sigma-Aldrich | S9251 |
| Phosphoric acid | Merck | 1.00573.1000 |
| Tumor necrosis factor alpha ELISA kit | Invitrogen | 176448001 |
| Interleukin 10 ELISA kit | Invitrogen | 218449-002 |
| LXRb ELISA kit | ELISAGenie (London, UK) | MOEB0190 |
| Estrogen receptor alpha | Elabscience (Wuhan, Hubei, PR China) | E-EL-M0476 |
| Estrogen receptor beta | Elabscience | E-EL-M0490 |

**Table S2.** Primer sequences for RT-qPCR

| **Gene** | **Primer forward** | **Primer reverse** |
| --- | --- | --- |
| *Tnf* | CATCTTCTCAAAATTCGAGTGACAA | TGGGAGTAGACAAGGTACAACCC |
| *Cxcl2* | AGTGAACTGCGCTGTCAATGC | AGGCAAACTTTTTGACCGCC |
| *Cd68* | TGACCTGCTCTCTCTAAGGCTACA | TCACGGTTGCAAGAGAAACATG |
| *Itgam* | ACTTTCAGAAGATGAAGGAGTTTGTCT | TGTGATCTTGGGCTAGGGTTTC |
| *Icam1* | CTACCATCACCGTGTATTCGTTTC | CGGTGCTCCACCATCCA |
| *Il18* | GACTCTTGCGTCAACTTCAAGG | CAGGCTGTCTTTTGTCAACGA |
| *Il1b* | AAAGAATCTATACCTGTCCTGTGTAATGAAA | GGTATTGCTTGGGATCCACACT |
| *Lxrb* | AAGCAGGTGCCAGGGTTCT | GTTTCTAGCAACATGATCTCAATGGT |

**Table S3.** Population characteristics of the Maastricht cohort

|  | **Females** | **Males** |
| --- | --- | --- |
| *n* | 21 | 13 |
| Age (years) | 43.76 ± 2.16 | 45.62 ± 2.33 |
| BMI (kg/m^2^) | 45.03 ± 1.68 | 44.70 ± 2.47 |
| Waist/hip ratio | 0.98 ± 0.06 | 1.11 ± 0.02 |
| Plasma total cholesterol (mg/dl) | 184.3 ± 9.82 | 159.3 ± 6.60 |
| Plasma HDL (mg/dl) | 39.22 ± 3.32 | 34.03 ± 2.87 |
| Plasma LDL (mg/dl) | 138.9 ± 6.13 | 113.7 ± 10.77* |
| Plasma triglycerides (mg/dl) | 187.7 ± 25.67 | 142.2 ± 27.06 |
|  |  |  |

Data are presented as mean ± SEM.

**p* ≤ 0.05 compared with females by use of two-tailed unpaired *t-*test.

**Table S4.** Characteristics of 7-week-old *Npc1^nih^* mice

|  | **Females** | **Males** |
| --- | --- | --- |
| *n* | 4 | 4 |
| Total weight (g) | 10.73 ± 0.80 | 12.43 ± 0.93 |
| Relative liver weight | 0.08 ± 0.002 | 0.09 ± 0.006 |
| Relative spleen weight | 0.006 ± 0.001 | 0.007 ± 0.001 |
| Plasma total cholesterol (mg/dl) | 102.0 ± 1.56 | 112.8 ± 17.58 |
| Plasma triglycerides (mg/dl) | 26.76 ± 3.35 | 43.35 ± 11.36 |
| Liver total cholesterol (mg/dl) | 0.02 ± 0.001 | 0.03 ± 0.001 |
| Liver total triglycerides (mg/dl) | 0.005 ± 0.0008 | 0.007 ± 0.0005 |
|  |  |  |

Data are presented as mean ± SEM.
